# Differential diagnosis of restlessness in a middle-aged woman from akathisia to frontotemporal dementia: A Case Presentation

**DOI:** 10.1192/j.eurpsy.2025.1762

**Published:** 2025-08-26

**Authors:** S. Turkan, R. S. İlhan, M. C. Saka

**Affiliations:** 1 Psychiatry, Ankara University, Ankara, Türkiye

## Abstract

**Introduction:**

Frontotemporal dementia (FTD) is the second most common cause of early-onset dementia and is clinically characterised by progressive behavioural changes, executive dysfunction and language difficulties. FTD is often confused with Alzheimer’s disease and other psychiatric disorders. Clinical features of FTD include personality changes, agitation, loss of inhibition, apathy, social withdrawal and impulsivity. In some cases, the disease is accompanied by mood or psychotic symptoms, resulting in the diagnosis of an additional psychiatric disorder. (Gliebus G.(2014). *SAGE open medical case reports*, *2*, 2050313X13519977.). This article presents the case of a middle-aged woman who was diagnosed with an anxiety spectrum disorder before developing and being diagnosed with FTD.

**Objectives:**

A 57-years-old right handed female with previous history of anxiety disorder admitted to psychiatry clinic with restlessness, decreased sleep, and complain of constant non-purpose walking. Physical examination revealed bradymimia and grabellar reflex. Additionally in her psychiatric evaluation she had short-term memory impairment, disinhibition and verbal perseverations. She had been given multiple combinations of medications by outpatient providers and her restlessness only increased. At the time of admission she was taking mirtazapine,olanzapine and clonazepam. The initial impression was that she had akathisia, and her medications were tapered. She was then started on propanolol and lorazepam. After several days her symptoms had not changed.

**Methods:**

In routine biochemical and hematological tests, electroencephalogram (EEG) were within normal limits. 18F-FDG PET/MRI revealed hypometabolism in the bilateral temporal-frontoparietal region, more pronounced in the frontal region which is consistent with FTD.

**Results:**

Trazodone was started to control behavioural symptoms and the dose was gradually increased to 150 mg/day. The dose of propranolol was increased to 80 mg/day, and lorazepam was tapered and discontinued. During the follow up with this treatment, there was an improvement in her restlessness and anxiety symptoms, but her memory problems were persistent.

**Conclusions:**

Frontotemporal dementia may overlap and be confused with other psychiatric disorders. Therefore, a comprehensive history, physical and neurological examination are required to differentiate each clinical entity (Khan I, De Jesus O. (2023).Frontotemporal Lobe Dementia. In: StatPearls ). Additionally, the use of functional neuroimaging, such as 18F-FDG PET/MRI, enables the different distribution of pathology in dementing disorders to be highlighted, as can be seen in our case. This case report highlights the importance of re-evaluating patients with psychiatric diseases, especially when symptoms are resistant to treatment.

**Disclosure of Interest:**

None Declared

